# Incidence and risk factors for AIDS-related mortality in HIV patients in China: a cross-sectional study

**DOI:** 10.1186/1471-2458-14-831

**Published:** 2014-08-11

**Authors:** Hui Zheng, Lu Wang, Peng Huang, Jessie Norris, Qing Wang, Wei Guo, Zhihang Peng, Rongbin Yu, Ning Wang

**Affiliations:** Department of Epidemiology and Biostatistics, School of Public Health, Nanjing Medical University, Nanjing, Jiangsu 211166 China; National Center for AIDS/STD Control and Prevention, Chinese Center for Disease Control and Prevention, Beijing, China

**Keywords:** HIV/AIDS, Highly active antiretroviral treatment, Mortality, China

## Abstract

**Background:**

To estimate the incidence and risk factors for mortality in HIV-1-infected patients in China.

**Methods:**

Information on AIDS-related deaths was collected from the Chinese Center for Disease Control and Prevention’s *Disease Surveillance Information Reporting System* and *AIDS Prevention and Control Information System*.

**Results:**

A total of 379,348 HIV cases were recorded in the databases from 2006. Among those, 138,288 patients were reported as having developed AIDS and 72,616 (19%) died of AIDS after data was extracted from the databases in January 2011. Mortality was higher among those patients aged 50 years old or older (AOR: 3.41, CI: 1.47-7.91) who had been infected by intravenous drug use (AOR: 1.65, CI: 1.28-2.14) or blood transfusion/donation (AOR: 2.18: 1.18-3.99). Compared to patients who had not initiated highly active antiretroviral therapy (HAART), those who had initiated HAART were more likely to have a long interval of time between infection confirmation and AIDS-related death.

**Conclusions:**

The effective reduction of AIDS mortality could be improved through timely treatment.

## Background

There are currently an estimated 780,000 people living with HIV in China; at the end of 2011, the cumulative number of reported deaths since the beginning of the HIV epidemic was 93,000 [1]. A report released by the Ministry of Health in 2009 showed that HIV/AIDS had become the leading cause of death from infectious disease in China [2].

The expansion of highly active antiretroviral therapy (HAART) in recent years has offered a hope of preventing thousands of deaths [3–12]. HAART [13] use has notably reduced HIV-related morbidity and mortality in both industrialized and low-income settings since 1996 [4, 14–17]. HAART has also contributed additional benefits such as prolonged disease-free survival, durable HIV virologic suppression, immunologic (CD4 cell) repletion, and reductions in hospitalization rates [14, 18, 19]. As a result of the Chinese government scaling up the National Free Antiretroviral Treatment Programme (NFATP) in 2003, approximately 10,000 people in China were receiving HAART by the end of 2011 [20].

Most previous reports in China have focused on the effects of HAART on clinical and immunologic outcomes compared to those observed in resource-rich settings [21]. Previous studies have already identified that patients starting HAART with low CD4 cell counts have fewer opportunistic infections such as tuberculosis, acute sepsis, cryptococcosis, and toxoplasmosis [22–25]. However, in-depth analysis and systematic research into causes of death among people with HIV/AIDS in China is lacking.

This study presents data from the China Disease Prevention and Control Information System on changes in AIDS mortality through December 31, 2010. It also determines the incidence and related risk factors of mortality following the widespread availability of HAART in China after 2003.

## Methods

### Data collection

Data on HIV/AIDS patients were collected from the *China Disease Prevention and Control Information System*. For mortality cases, detailed information was downloaded from the *Disease Surveillance Information Reporting System* and *AIDS Prevention and Control Information System*. These databases contain information on patient demographic characteristics, reported provinces of residence, survival time (from diagnosis to death), cause of death, and HAART use. If one case was contained in both databases, we selected the information on the case from *AIDS Prevention and Control Information System.* All data were assessed from January 1, 2006 to December 31, 2010. HAART was scaled up nationwide in 2003, with data prospectively included in the database after 2004. However, the data collected from 2004 to 2005 covered only part of the provinces, and nationwide collection was not yet initiated; for this reason, the mortality data did not start until 2006.

All records in the databases had undergone quality control, and cases were confirmed in a laboratory. Data were recorded solely for individuals from mainland China, excluding people from Hong Kong, Macao and Taiwan. Non-Chinese citizens were also excluded from our study.

A second analysis was conducted on mortality cases from January 1, 2011 to July 31, 2011. The effects of treatment on death were analyzed by studying the number of days between the date of death and the date of either the first CD4 test or the date of AIDS diagnosis if CD4 test results were unavailable.

### Ethics statement

This study, including design, recruitment, consent, and assessment procedures, was reviewed and approved by the Institutional Review Board of Nanjing Medical University.

### Cause of death

Causes of death for cases reported in the *Data Information Management System* were verified. Among the 32,562 cases with a cause of death listed as “other reasons”, some were confirmed as AIDS-related death by verifying the information filed. When the cause of death was listed as tuberculosis, opportunistic infections (OI), pneumocystis pneumonia (PCP), or AIDS-related disease, the cause of death was reclassified as AIDS. Taking into account that tuberculosis is prevalent in China, those deaths were removed from the analysis of AIDS-related deaths if the patient’s last CD4 did not fall below 200/uL, to avoid overestimating the number of AIDS-related deaths.

### Identify key variables

A late diagnosis of AIDS-related death was defined as a diagnosis of AIDS (CD4 < 200 cells/μL or AIDS-related clinical symptoms) [26] where death occurred within one year after an HIV diagnosis. All patients were eligible for free treatment if their HIV was in WHO clinical stage 3 or 4, or if they had a CD4 count < 200 cells/μL or <350 cells/μL after 2008. Untreated deaths were defined as patients who died without initiating treatment. Mortality cases with antiviral treatment information in the treatment database were matched to the same cases in the mortality database. The deaths of patients who were not listed in the treatment registry were considered untreated deaths.

### Statistical analysis

SPSS (version 20.0), Stata (version 12.0) and Excel (version 2010) were used for data analysis. Descriptive analyses were conducted to describe mortality cases’ characteristics, including mean (±SD), median (interquartile range, IQR), and frequencies (%). A logistic regression was applied to determine the risk factors of HIV/AIDS mortality. Kaplan-Meier survival curves were used to estimate the probability of death and the median time to death after HAART initiation. The log-rank test was used to compare the median time to death between the four groups. Statistical significance was assessed at the 0.05 level, and all hypothesis tests were two-sided.

## Results

### General characteristics

In our study, annual reported deaths from HIV/AIDS from 2006 to 2010 comprised 1809; 5544; 9748; 12,287; and 18,987 cases, respectively. The estimated annual number of new infections was 70,000; 50,000; 48,000; and 48,000 in 2005, 2007, 2009, and 2011, respectively. Based on our data, we estimated the annual mortality rates as being 2.5% in 2007, 3.5% in 2008 and 5.0% in 2010. The actual annual number of HIV/AIDS deaths after verifying cause of death from 2006 to 2010 was 7,013; 9,298; 11,921; 13,832; and 13,981, respectively, revealing a slower increase than in the data reported. Annual cases of AIDS-related deaths among actual annual cases from 2006 to 2010 were 2764; 3575; 4877; and 5675; and 5,793, respectively, showing a relatively slight upward trend.

The total number of deaths in male patients was 54,904. Among all patients, death was more common among those who were 20–49 years of age (75.3%). The median age of death was 38.5 years old, 38.4 years for males and 38.7 years for females. Among self-reported sources of transmission, the proportions of total deaths from infections through heterosexual transmission, injection drug use (IDU), blood transfusion/donation, homosexual transmission and others were 31.7%, 28.4%, 24.0%, 0.8% and 1.1%, respectively. One-fifth (19.7%) of HIV-infected patients among cumulative deaths reported the use of HAART (Table 1).

**Table 1:** **Demographics of the actual HIV/AIDS deaths and their associated risk factors from 2006 to 2010**

Year	2006	2007	2008	2009	2010	Total No. (%)	OR (95% CI)	AOR (95% CI)
Death	7013	9298	11921	13832	13981	72616		
**Sex**								
Male	5167	6974	9013	10688	10874	54904 (75.6)	1.0	1.0
Female	1846	2324	2908	3144	3107	17712 (24.4)	1.51 (1.22-1.87)	1.02 (0.79-1.32)
**Age (median)**	37	38	39	40	41	38.5	1.05 (1.04-1.06)	1.03 (1.01-1.06)
0-20	170	226	210	231	214	1624 (2.3)	1.0	1.0
20-29	1199	1455	1632	1667	1589	11153 (15.4)	0.85 (0.41-1.78)	0.89 (0.40-1.97)
30-39	2817	3690	4526	4870	4597	27183 (37.4)	1.66 (0.81-3.41)	1.34 (0.61-3.00)
40-49	1582	2028	2755	3195	3466	16365 (22.5)	2.02 (0.97-4.21)	1.39 (0.61-3.19)
>50	1244	1899	2798	3869	4115	16188 (22.3)	5.33 (2.55-11.15)	3.41 (1.47-7.91)
**Transmission routes**								
Heterosexual	1423	2509	4264	5974	6796	23009 (31.7)	1.0	1.0
IDU	1872	2729	3402	3784	3942	20616 (28.4)	1.36 (1.11-1.68)	1.65 (1.28-2.14)
Blood transfusion/donation	2288	2178	2154	1513	1495	17487 (24.0)	3.34 (1.98-5.61)	2.18 (1.18-3.99)
Homosexual	22	48	96	151	275	615 (0.8)	0.18 (0.14-0.25)	0.33 (0.23-0.47)
Others	94	133	113	146	127	792 (1.1)	0.77 (0.50-1.20)	0.87 (0.48-1.56)
**Marital status**								
Unmarried	1386	1997	2479	2843	2935	14547 (20.0)	1.0	1.0
Married	4260	5395	7093	8071	7938	42239 (58.2)	2.43 (1.99-2.97)	1.16 (0.89-1.50)
Divorced or widowed	743	1171	1536	2128	2378	9109 (12.5)	2.53 (1.95-3.28)	1.12 (0.81-1.56)
**Received HAART**								
No	5380	7423	8945	10856	10866	58318 (80.3)	1.0	1.0
Yes	1633	1875	2513	2976	3115	14298 (19.7)	0.41 (0.32-0.51)	0.11 (0.08-0.14)

The three risk factors most strongly related to mortality were age, not having received HAART and having multiple transmission routes. IDUs (AOR, adjusted odds ratio: 1.65, CI, confidence interval: 1.28-2.14) and blood transfusion/donations (AOR: 2.18, CI: 1.18-3.99) were significantly more likely to having a high mortality compared to those infected through heterosexual transmission; however, the statistical results of individuals infected by homosexual transmission (AOR: 0.33, CI: 0.23-0.47) were just the opposite. Patients aged 50 years old or older (AOR: 3.41, CI: 1.47-7.91) and those who had not received HAART were also significantly likely to have a high mortality. Although sex and marital status were not significantly associated with mortality, female gender and a status of married or divorced had a high risk of mortality in the unadjusted analysis, but these factors were not included in the adjusted analysis (Table 1).

AIDS deaths were reported in the 31 mainland provinces by the end of 2010. The top six provinces with the highest cumulative number of reported HIV/AIDS cases, as well as the highest cumulative number of reported deaths, were Yunnan, Guangxi, Henan, Sichuan, Xinjiang and Guangdong. The top five provinces with the highest proportion of cumulative reported deaths accounting for the cumulative number of reported HIV/AIDS cases were Shanxi, Hubei, Henan, Hebei, and Anhui. The proportion of HIV/AIDS-related deaths was low in provinces such as Beijing, Shanghai, Tianjin, and Zhejiang (Table 2).

**Table 2:** **The provincial statistics of the cumulative reported cases and the cumulative AIDS-related death cases with HAART**

Province	Cumulative reported cases			Cumulative death cases			Proportion of deaths (%)
	Number	Accepted HARRT	Percentage (%)	Number	Accepted HARRT	Percentage (%)	
Anhui	6590	3564	54.1	1730	446	25.8	26.3
Beijing	5259	1053	20	148	25	16.9	2.8
Fujian	2649	710	26.8	568	55	9.7	21.4
Gansu	893	234	26.2	163	19	11.7	18.3
Guangxi	63127	16560	26.2	1359	1329	9.8	21.5
Guangdong	28534	4471	15.7	5829	437	7.5	20.4
Guizhou	10290	1351	13.1	1826	143	7.8	17.7
Hainan	975	98	10.1	292	9	3.1	29.9
Hebei	1913	754	39.4	607	110	18.1	31.7
Henan	49325	32282	65.4	1467	7471	50.9	29.7
Heilongjiang	1571	412	26.2	228	34	14.9	14.5
Hubei	6613	2689	40.7	2039	439	21.5	30.8
Hunan	10794	2736	25.3	2831	374	13.2	26.2
Jilin	1504	479	31.8	387	66	17.1	25.7
Jiangsu	4084	1337	32.7	643	102	15.9	15.7
Jiangxi	2556	983	38.5	822	169	20.6	32.2
Liaoning	2785	585	21	347	49	14.1	12.5
Neimengu	621	119	19.2	97	20	20.6	15.6
Ningxia	370	77	20.8	44	10	22.7	11.9
Qinghai	317	98	30.9	45	11	24.4	14.2
Shandong	2272	754	33.2	547	87	15.9	24.1
Shanxi	3055	1180	38.6	1002	158	15.8	32.8
Shanxi	1569	460	29.3	309	72	23.3	19.7
Shanghai	5260	1154	21.9	239	48	20.1	4.5
Sichuan	38356	5041	13.1	5485	440	8	14.3
Tianjin	885	237	26.8	99	22	22.2	11.2
Tibet	126	8	6.3	10	1	10	7.9
Xinjiang	33519	4119	12.3	4107	379	9.2	12.3
Yunnan	79433	18953	23.9	1222	1537	12.6	15.4
Zhejiang	4713	1695	36	494	88	17.8	10.5
Chongqing	9390	1603	17.1	1179	148	12.6	12.6
Nationwide	379348	105796	27.9	72616	14298	19.7	19.1

### Characteristics according to time since HIV diagnosis and HAART

Among the 72,616 deaths, the median time between diagnosis and death was 0.7 years (IQR: 0.1, 2.6). The median time of HIV infection between diagnosis and death was 0.9 years (IQR: 0.1, 2.9). Simultaneously, the median time of AIDS infection between diagnosis and death was 0.6 years (IQR: 0.1, 2.4).

The median time between diagnosis and death for patients receiving treatment was 1.6 years (IQR: 0.44, 3.62), while the median time between diagnosis and death for untreated patients was 0.5 years (IQR: 0.1, 2.2).

Table 3 presents and compares the interval between diagnosis and death according to HAART initiation status. The different day ranges between diagnosis and death have different numbers of death, more than 180 days was the most, less than 30 days, 30 to 60 days, 60 to 90 days, 90 to 180 days was 15615, 5543, 4002, 7164, respectively. Among all deaths by the end of 2010, 80.3% (58318/72616) of patients who died did not receive HAART and 75.0% (43732/58318) of those had no CD4 test result. For those patients with CD4 test results, 62.8% (9167/14586) had a CD4 count below 200 at their first lab test, and 78.9% (11643/14586) had a CD4 count below 350 at their first lab test. Compared to patients who had not initiated HAART, those who had initiated HAART were more likely to have a long interval of time between diagnosis and death (*χ*^*2*^ = 3621.19, *p* < 0.001).

**Table 3:** **HAART and CD4 test results of the cumulative HIV/AIDS deaths by the end of 2010**

The interval between infection confirmation and deaths (day)	Deaths	Initiated HAART No. (%)	Not receiving HAART				
			First laboratory CD4 count				No CD4 test result No. (%)
			≤200	200-350	>350	Total No. (%)	
<30	15615	610 (3.9)	1346	77	61	1484 (9.5)	13521 (86.6)
30-	5543	788 (14.2)	1014	77	54	1145 (20.7)	3610 (65.1)
60-	4002	832 (20.8)	831	52	60	943 (23.6)	2227 (55.6)
90-	7164	1573 (22.0)	1400	161	170	1731 (24.2)	3860 (53.9)
≥180	40292	10495 (26.0)	4576	2109	2598	9283 (23.0)	20514 (50.9)
Total	72616	14298 (19.7)	9167	2476	2943	14586 (20.0)	43732 (60.2)

According to the *AIDS Network Reporting System,* by the end of 2010, 56.1% (40746/72616) of the cumulative number of patients who died were late diagnoses. Of those who died after receiving a late diagnosis, 73.5% were male, 64.6% were married, and 74.5% were ethnically Han. Transmission categories for these deaths included heterosexual transmission (43.4%), IDU (15.9%), blood donation (14.4%), and blood transfusion (8.3%). Most (61.9%) patients were first diagnosed in their counties of residence.

Among the patients who died who had never initiated HAART, 77.4% were male, 55.5% were married, and 67.3% were ethnically Han. Transmission categories included heterosexual transmission (33.2%), IDU (32.0%), blood donation (11.3%) and blood transfusion (5.4%). Most (60.9%) patients were first diagnosed in their counties of residence (Table 4).

**Table 4:** **Demographics of late diagnosis of HIV deaths and untreated deaths by the end of 2010**

	Late diagnosis of deaths	Untreated deaths cases
	No. (%)	No. (%)
Sex		
Male	29954 (73.5)	45162 (77.4)
Female	10792 (26.5)	13155 (22.6)
Age (years)		
0-9	1062 (2.6)	634 (1.1)
10-19	/	533 (0.9)
20-29	5556 (13.6)	6339 (10.9)
30-39	13709 (33.7)	13795 (23.7)
40-49	9360 (23.0)	8801 (15.1)
50	10981 (27.0)	10567 (18.1)
Marital status		
Married	26336 (64.6)	32390 (55.5)
Unmarried	7019 (17.2)	12518 (21.5)
Divorced or widowed	5222 (12.8)	6913 (11.9)
Ethnicity		
Han	30373 (74.5)	39220 (67.3)
Zhuang/Wei/Yi/Dai	5352 (13.1)	10078 (17.3)
Other ethnicity	/	/
Infection Routes		
Heterosexual	17693 (43.4)	19387 (33.2)
IDU	6462 (15.9)	18666 (32.0)
Blood Donation	5847 (14.4)	6606 (11.3)
Blood Transfusion	3391 (8.3)	3158 (5.4)
MTCT	510 (1.3)	677 (1.2)
MSM	453 (1.1)	487 (0.8)
Sex (both MSM and heterosexual ) + IDU	283 (0.7)	452 (0.8)
Unknown	/	8371 (14.4)
Location of death		
Local county	25309 (61.9)	35523 (60.9)
Local city and other county	8116 (19.9)	10671 (18.3)
Local province and other city	5507 (13.5)	9258 (15.9)
Other province	1914 (4.7)	2865 (4.9)
Reporting units		
Disease control system	24833 (61.0)	39862 (68.4)
Medical institution	15744 (38.6)	17746 (30.4)
Blood center	26 (0.1)	105 (0.2)
Drug addiction treatment facility	53 (0.1)	472 (0.8)
Occupation		
Farmer	24411 (59.9)	31839 (54.6)
Housekeeper and unemployed	4556 (11.2)	9039 (15.5)
Laborer	2083 (5.1)	2784 (4.8)
Retired	1392 (3.4)	1528 (2.6)
Migrant worker	1308 (3.2)	1522 (2.6)
Business	/	1346 (2.3)

### Underlying cause of death

Out of 72,616 total deaths, there were 63,785 cases with information on cause of death, of which 31,223 (49.0%) were considered AIDS-related deaths and 32,562 had other causes of death. AIDS deaths accounted for 57.1% of all deaths after cause of death was verified. Results are shown in Table 5.

**Table 5:** **Cause of death for HIV cases by the end of 2010**

Causes of death	Original report		After adjustment	
	Number of cases	%	Number of cases	%
AIDS	31223	49.0	36438	57.1
Other	32562	51.1	27347	42.9
Total	63785	100.0	63785	100.0

### Characteristics of treated deaths by baseline CD4 cell counts

Based on the data collected from January 1, 2011 to July 31, 2011, most of the patients who died without initiating HAART died either more than 12 months from diagnosis (n = 2,027) or less than three months from diagnosis (n = 2,543). More than half (57.5%) never received a CD4 test before their death. Among the 2,375 patients who had received a CD4 test, 1,772 had first CD4 test results that met the criteria to initiate treatment. Among 603 patients with CD4 counts >350 cells/μL, 210 (34.8%) had a subsequent CD4 test with a count < 350 cells/μL (Table 6).

**Table 6:** **CD4 test results for mortality cases from January 1, 2011 to July 31, 2011**

Interval between death and confirming (months)	Number of deaths	Proportion with at least one CD4 test N (%)	First CD4 test results			
			<50	50-200	201-350	>350
0-2	2543	567 (22.3%)	360	145	28	34
3-5	457	245 (53.6%)	124	83	18	20
6-8	334	189 (56.6%)	82	62	15	30
9-11	222	120 (54.1%)	33	47	11	29
≥12	2027	1254 (61.9%)	173	274	317	490
Total	5583	2375 (42.5%)	772	611	389	603

**Figure 1:**
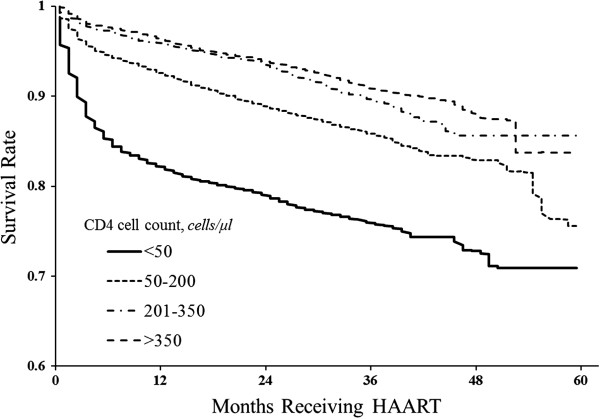
**The survival probability for cases receiving antiretroviral therapy, arranged by baseline CD4 cell count.**

The probability of survival after receiving HAART is shown by the Kaplan-Meier method in Figure 1. The study population was analyzed in four groups based on baseline CD4 cell counts (Group 1: CD4 count <50 cells/μL, Group 2: CD4 count ≥50 cells/μL and ≤200 cells/μL, Group 3: CD4 count >200 cells/μL and ≤350 cells/μL, and Group 4: CD4 count > 350 cells/μL). Survival rates at 12, 36, and 60 days were 82.1%, 75.9%, and 70.9% in Group 1; 92.6%, 85.8%, and 82.7% in Group 2; 95.9%, 89.6% and 85.6% in Group 3; and 96.4%, 90.8% and 87.4% in Group 4 (log-rank test, *p* <0.001).

## Discussion

This study showed some notable findings on causes of death among a large and demographically diverse population of HIV patients. Mortality was higher among those patients aged 50 years old or older who had been infected by IDU or blood transfusion/donation and had not accepted HAART.

According to our study, the risk factor strongly related to death was age, especially for individuals aged 50 years old or older. There may be many reasons for this. First of all, a substantial proportion of elderly people who meet treatment criteria may give up treatment voluntarily. This is likely to be associated with the personal characteristics of this group, such as having low levels of education and desiring to reduce the burden on family. Furthermore, individuals in older age groups may be likely to refuse treatment as a result of the stigma associated with HIV/AIDS [27], without the support of social and family members. Last but not least, elderly people may be underserved by the public health system, especially in some less developed areas.

The study noted a significant risk of mortality in individuals who were infected by IDU and blood transfusion/donation compared to heterosexual transmission. IDU was the primary mean of transmission at the beginning of China’s HIV epidemic [28], which led to a long period of infection and late treatment initiation. Consequently, those infected by IDU had a higher mortality rate, which is consistent with other reports [29, 30]. In addition, the high mortality in IDUs may be associated with personal characteristics; some foreign studies indicated that the majority of accidental deaths among IDUs are from drug overdoses [31, 32].

Similarly, blood transfusion was also one of the main routes in the early phase of China [33], most former plasma donors (FPD) or blood receptors infected by HIV long time ago, which made the disease developed long enough to become a danger for bodies and thus a risk factor for higher mortality [34].

Significantly, there is a protective influence in individuals infected by homosexual transmission, in contrast with individuals infected by heterosexual transmission. There may be a bias here due to the database’s small sample of men who have sex with men (MSM), although the population of MSM has begun to increase in recent years in China [35, 36]. Another potential reason may be that the majority of MSM are relatively young.

The results of our study reveal a statistically significant difference between accepting HAART or not. The results provide evidence that increasing HAART coverage at the population level can decrease HIV-related mortality, which conforms with the results of overseas findings such as those from the mid-to-late 1990s in the USA [4], Europe [37] and in other earlier studies [38–40].

In the five provinces with the highest proportion of cumulative AIDS deaths, patients who died almost always had a history of paid blood donation. These regions excelled at early detection, management of cases and follow-ups, and reporting deaths. Conversely, in Beijing, Shanghai, Tianjin, and Zhejiang, the proportion of cumulative AIDS deaths was the lowest among all provinces. The reason for the low AIDS deaths in these areas may be due to their large migrant populations, whose high mobility complicates follow-up.

According to previous studies, end-stage patients with low CD4 counts achieve significantly fewer life-prolonging effects through HAART than those with high CD4 counts. However, according to the results of this study, most of those who died before initiating HAART never had a CD4 test. Efforts should be made to improve coverage of HIV diagnostic tests and the frequency of CD4 testing in order to offer timely HAART to prolong survival time. The median time between diagnosis and death was only 0.7 years, and nearly half of cases were discovered late. Though HAART can effectively reduce the fatality rate of HIV/AIDS, many at-risk individuals do not seek out standard HIV counseling and testing services. The stigmas associated with drug use and HIV/AIDS and the fear of arrest or of a positive result can be major barriers to accessing HIV voluntary counseling and testing (VCT) services [41]. It is critical to scale up early monitoring to provide prompt treatment and effectively reduce AIDS mortality.

The results of this survival analysis indicate the benefits of HAART in reducing overall mortality and AIDS-related morbidity, which is similar to results in other studies [42, 43]. However, the cumulative number of HIV-positive adults using HAART in China was less than 20% by the end of 2010 [20]. The results indicate that HIV-positive individuals need to be diagnosed much earlier, which would suggest that HIV testing programs should be expanded.

In our study, survival analysis in HIV patients received HAART also indicated that individuals were more likely to have a long interval of time between diagnosis and death compared to individuals who had high baseline CD4 cell counts. The differences were more visible between Group 1 and Group 4. Timely HAART should be provided to prolong survival time, as receiving HAART is the best way to reduce mortality. Mechanisms should be in place to prevent the development of drug resistance and to enhance clinical services, including implementing viral load testing, increasing adherence, and providing prompt second-line therapy for patients with first-line treatment failure.

This study had several limitations. First, data were used from sentinel detection databases and may not be representative of all deaths in China, may exclude those who were homeless or living alone when they died and may underestimate AIDS-related mortality. Second, many could have died of AIDS, but if they were never diagnosed the cause of death could have been listed as something else and they would not be included in the databases. Third, data may have been missing from the databases for other reasons. Missing data may influence the determination of receiving HAART or not, which likely underestimates the proportion of patients who had initiated HAART.

## Conclusions

In summary, early diagnosis of HIV can maximize the effectiveness of HAART. It is essential to continue monitoring HAART uptake and adherence in China, which will help to save lives.

## Abbreviations

HAART: Highly active antiretroviral therapy
NFATP: National Free Antiretroviral Treatment Programme
OI: Opportunistic infections
PCP: Pneumocystis pneumonia
IDU: Injection drug use
MSM: Men who have sex with men
VCT: Voluntary counseling and testing.
